# Clinical and biological role of secretory phospholipase A2 in acute respiratory distress syndrome infants

**DOI:** 10.1186/cc12842

**Published:** 2013-07-24

**Authors:** Daniele De Luca, Elena Lopez-Rodriguez, Angelo Minucci, Francesca Vendittelli, Leonarda Gentile, Eleonora Stival, Giorgio Conti, Marco Piastra, Massimo Antonelli, Mercedes Echaide, Jesus Perez-Gil, Ettore D Capoluongo

**Affiliations:** 1 Pediatric Intensive Care Unit, Dept of Anesthesiology and Intensive Care, University Hospital 'A. Gemelli', Catholic University of the Sacred Heart, L.go A. Gemelli 8, 00168 Rome, Italy; 2 Laboratory of Clinical Molecular Biology, Dept of Laboratory Medicine, University Hospital 'A. Gemelli', Catholic University of the Sacred Heart, L.go A. Gemelli 8, 00168 Rome, Italy; 3 Dept of Biochemistry, Faculty of Biology, Complutense University, av. Complutense s/n, 28040 Madrid, Spain

## Abstract

**Introduction:**

Secretory phospholipase A2 is supposed to play a role in acute lung injury but no data are available for pediatric acute respiratory distress syndrome (ARDS). It is not clear which enzyme subtypes are secreted and what the relationships are between enzyme activity, biophysical and biochemical parameters, and clinical outcomes. We aimed to measure the enzyme and identify its subtypes and to study its biochemical and biophysical effect. The secondary aim was to correlate enzyme activity with clinical outcome.

**Methods:**

Bronchoalveolar lavage was performed in 24 infants with ARDS and 14 controls with no lung disease. Samples were assayed for secretory phospholipase A2 and molecules related to its activity and expression. Western blotting and captive bubble surfactometry were also performed. Clinical data were real time downloaded.

**Results:**

Tumor necrosis factor-α (814 (506-2,499) *vs*. 287 (111-1,315) pg/mL; *P *= 0.04), enzyme activity (430 (253-600) *vs*. 149 (61-387) IU/mL; *P *= 0.01), free fatty acids (4.3 (2.8-8.6) *vs*. 2 (0.8-4.6) mM; *P *= 0.026), and minimum surface tension (25.6 ± 6.1 *vs*. 18 ± 1.8 mN/m; *P *= 0.006) were higher in ARDS than in controls. Phospholipids are lower in ARDS than in controls (76.5 (54-100) *vs*. 1,094 (536-2,907) μg/mL; *P *= 0.0001). Three enzyme subtypes were identified (-IIA, -V, -X), although in lower quantities in controls; another subtype (-IB) was mainly detected in ARDS. Significant correlations exist between enzyme activity, free fatty acids (ρ = 0.823; *P *< 0.001), and surface tension (ρ = 0.55; *P *< 0.028). Correlations also exist with intensive care stay (ρ = 0.54; *P *= 0.001), PRISM-III_24 _(ρ = 0.79; *P*< 0.001), duration of ventilation (ρ = 0.53; *P *= 0.002), and oxygen therapy (ρ = 0.54; *P *= 0.001).

**Conclusions:**

Secretory phospholipase A2 activity is raised in pediatric ARDS and constituted of four subtypes. Enzyme correlates with some inflammatory mediators, surface tension, and major clinical outcomes. Secretory phospholipase A2 may be a clinically relevant target in pediatric ARDS.

## Introduction

Secretory phospholipase A2 (sPLA2; EC: 3.1.1.4) belongs to an ubiquitous enzyme superfamily, crucial for the inflammation pathway [1]. In fact, sPLA2 releases free fatty acids (FFA) from the *sn-2 *position of phospholipids, producing arachidonic acid and its derivatives. Over 10 distinct sPLA2 isotypes carrying different substrate specificity have been described in mammalians and four of these (sPLA2-IB, - IIA, -V, and -X) are expressed in total lung extracts [2]. sPLA2s are relevant in lung physiopathology, since they may affect pulmonary function, either producing inflammatory mediators or directly catabolizing surfactant through the hydrolysis of its phospholipids.

Previous studies have shown that the subtype -IIA of sPLA2 is increased in broncho-alveolar lavage fluid (BALF) in animal models of acute lung injury [3,4] and sPLA2-IIA levels seem to correlate with clinical severity in adults with acute respiratory distress syndrome (ARDS) [5,6]. Other sPLA2 subtypes expressed in the lung have been suspected to play a role in ARDS, however their presence in human BALF has never been studied [2].

ARDS in infants differs from the syndrome in adults in terms of epidemiology, triggering causes and prognosis [7], thus requires specific additional research [8]. Since sPLA2 is responsible for surfactant catabolism, its role is even more important in this context. In fact, surfactant replacement therapy has been tried, but it is not always beneficial in all infants [9]. This may be due to the inactivation by sPLA2, that creates a vicious cycle reducing surfactant efficiency [10]. Interestingly, several sPLA2 inhibitors carrying various specificities for sPLA2 subtypes, are now becoming available [11]. In a preliminary study on ARDS infants (conducted in 2007-2008), high levels of sPLA2 and significant correlations with oxygenation impairment and clinical severity have been found [12]. Thus, targeting sPLA2 could be an intriguing strategy for ARDS, although a proof of concept of its clinical importance is still lacking.

This study was designed to fill this gap. Our main purposes were: (1) to measure sPLA2 and the molecules related to its expression and activity and to identify the enzyme subtypes secreted into the alveoli; (2) to study the biochemical and biophysical effects of sPLA2 in BALF of infants with ARDS. The secondary aim was to correlate sPLA2 levels with some clinical outcomes. This study is a part of an international project investigating the role of sPLA2 in various pediatric respiratory diseases, whose plan has been described elsewhere [13].

## Materials and methods

### Patients

Eligible babies were all infants aged >30 days and ≤12 months admitted to our pediatric intensive care unit (PICU) during 2011, diagnosed with ARDS, according to the American-European criteria [14]. All babies were subjected to echocardiography to exclude left atrial hypertension and heart failure; no patient received steroids. Controls fulfilled all the following criteria: (1) >30 days and ≤12 months of age; (2) intubation for reasons other than any lung disease; (3) normal chest X-rays and clinical examination; (4) PaO_2_/FiO_2_>300 or FiO_2 _= 0.21; (5) normal C-reactive protein; and (6) no respiratory diseases in the previous 3 months. Exclusion criteria for both groups were: (1) need for thoracic surgery; (2) congenital complex or lung malformations; and (3) extracorporeal life support. Enrolment took place within 6 h from the fulfilling of ARDS criteria or from intubation (in controls). Participation to the study did not modify in any way the routine clinical assistance: study protocol was approved by the ethical committee of the University Hospital 'A. Gemelli' and informed consent was given by parents. Twenty-four cases and 14 controls resulted eligible during the study and all were prospectively enrolled: study population is described in Table 1.

**Table 1:** Basic characteristics of study population.

	ARDS group (*n *= 24)	Control group (*n *= 14)	*P*
Age (months)	4 (2-10.5)	6.5 (2-10.5)	0.457

Weight (Kg)	5.5 (4-10.8)	7 (4.8-9.6)	0.796

Male sex	13 (54.2)	8 (57.1)	0.643

PRISM-III_24_	16.5 (8.5-20.5)	3 (2-5.5)	<0.001

Modified Murray's score	5 (4.2-6)	0.75 (0.5-1.1)	<0.001

PaO_2_/FiO_2_	127 (86-158)	510 (358-667)	<0.001

OI	12.7 (9.9-18.5)	1.7 (1.2-2.2)	<0.001

Crs (mL/cmH_2_O/kg)	0.35 (0.24-0.4)	0.9 (0.8-0.9)	<0.001

Deaths	3 (12.5)	1 (7.1)	0.627

Co-morbidities	Severe sepsis (5)	Metabolic disease (1)	
	RSV infection (4)	SAH (1)	
	H1N1 flu (4)	Status epilepticus (2)	
	Aspiration (3)	General anesthesia (10)	
	Smoke inhalation (4)		
	Trauma (2)		
	Late onset GBS infection (1)		
	Malignancy (1)		

Data are expressed as median (interquartile range) or number (%). Murray's score, PaO_2_/FiO_2_, OI and Crs are measured or calculated just before the bronchoalveolar lavage. Co-morbidities indicate the ARDS etiology or the reasons for intubation in controls.

ARDS: acute respiratory distress syndrome; Crs: respiratory system static compliance; GBS: Group B streptococcus; HFOV: high frequency oscillatory ventilation; OI: oxygenation index; RSV: respiratory sincitial virus; SAH: sub-arachnoid hemorrhage.

### Ventilation protocol

Infants were ventilated according to our formal routine protocol that was unchanged during the study. Such protocol is reviewed every year and all physicians agreed on it. Servo-*I *ventilators (Maquet, Solna, Sweden) and cuffed endotracheal tubes were used to ventilate both cases and controls. ARDS patients were ventilated using pressure-controlled time-cycled modality, targeted at a tidal volume (V_T_) of 6 mL/kg. Positive end-expiratory pressure (PEEP) was set between 6 and 15 cmH_2_O and FiO_2 _at the minimum level to obtain an arterial oxygen saturation (SatO_2_) of 90-95%. PaCO_2 _values causing pH >7.30 were allowed. Rescue high frequency oscillatory ventilation (HFOV) was started according to our formal protocol [15]: in detail, HFOV is promptly instituted when a plateau pressure ≤28 cmH_2_O did not allow to reach the desired V_T _or when it was not possible to keep SatO_2 _>90%, with a FiO_2 _≤0.6. Apnoeic sedation was provided with remifentanil (0.1-0.2 γ/Kg/min) and midazolam (1-2 γ/Kg/min). After the acute phase, ARDS patients were switched to pressure support ventilation when they had no hemodynamic or temperature instability and fulfilled the following criteria: mean airway pressure (Pāw) ≤15 cmH_2_O, FiO_2 _≤0.5 with a SatO_2 _constantly >90% and no desaturation >5%, while suctioning. Pressure support was targeted to obtain V_T _of 6 mL/kg and stepped down as long as the ARDS patient was improving. PEEP during pressure support was set between 5 and 8 cmH_2_O; flow trigger was set at the highest possible sensitivity without auto-cycling; cycling-off was set at the 50% of the peak inspiratory flow. ARDS patients were extubated to continuous positive airway pressure at the same level of PEEP (5-8 cmH_2_O). Extubation took place when they fulfil the following criteria: (1) SatO_2 _≥95% on PEEP ≤5 cm H_2_O and FiO_2 _≤50%; (2) minimal V_T _= 5 mL/kg; and (3) respiratory rate appropriate for the age [16]. In the control group, V_T_, PEEP, and FiO_2 _were 7-8 mL/kg, 4-5 cmH_2_O, and <0.25, respectively.

### Bronchoalveolar lavage

Non-bronchoscopic bronchoalveolar lavage was performed within 6 h from the fulfilling of ARDS criteria in patients or from the intubation in controls. Since this is a part of our routine protocol for microbiological surveillance, no procedure was done solely for the study purposes. Lavage was performed as already described [12] and following advices of the European Respiratory Society Pediatric Task Force [17]. In detail, broncho-alveolar lavage was performed by instillation of two sequential aliquots of 1 mL/kg (up to a maximum of 5 mL) 0.9% NaCl warmed at 37°C, into the endotracheal tube, followed by three respiratory cycles. A straight, snub-nosed, end-hole suction catheter was gently advanced into the endotracheal tube, while continuing ventilation through a Y-connector. When resistance was met, suctioning with 50 mmHg of negative pressure was applied. The procedure was repeated with the infant's head turned 90° to the left and then to the right in order to virtually ensure sampling from the right and the left lung, respectively. The first collected fluid, reflecting tracheo-bronchial *milieu*, was sent for microbiological culture. The second aliquot consisted on average of 1.8 ± 0.5 mL (about 40% of the instilled volume) and was diluted with 0.9% saline up to 2 mL and centrifuged (3,000 rpm; 4°C; 10'). Cell-free supernatants were separated, immediately frozen at -80°C and thawed only once for the experiments. All samples were sterile and did not show visible blood contamination.

### sPLA2 assay and identification in BALF

BALF supernatants were centrifuged (12,000 rpm; 10' and then 3,500 rpm; 3') through a membrane-filter with a molecular weight cutoff of 30 kDa (Amicon Ultra; Millipore, Billera, MA, USA), as previously published [18]. This aims at separating secretory and cytosolic phospholipases (which weigh approximately14 kDa and 80 kDa, respectively [2]). sPLA2 total activity was then measured with a non-radioactive method, as previously published [19]. Coefficient of variation and detection range were <5% and 5-80 IU/mL, respectively.

Different sPLA2 subtypes were identified by western blotting, as follows. BALF samples were lyophilized after trifluoroacetic acid treatment and 20 μg of their proteins were resuspended in phosphate-buffered saline and dye-sample loading buffer (50 mM Tris-HCl, pH 6.7; 10% glycerol; 3% SDS; 1% β-mercaptoethanol; 0.01% bromophenol blue). Samples were then boiled (100°C; 10'), centrifuged (450g; 5'; 4°C) and then loaded into the gels. Electrophoresis was performed using 12% SDS polyacrilamide and protein bands were later transferred into PVDF membranes (Millipore - Billerica, MA, USA) by semi-dry transfer system (Bio-Rad -Hercules, CA, USA), using standard protocols. After gentle shacking of 4 h in blocking solution (5% non-fat dry milk, PBS, and 0.5% Tween 20), blots were first incubated with anti-sPLA2-IB (polyclonal), -V (monoclonal), or -X (polyclonal) antibodies (Santa-Cruz Biotechnology, Santa Cruz, CA, USA). Anti-sPLA2-IIA (monoclonal) antibody (Cayman Chemical, Ann Arbor, MI, USA) was also used. All these antibodies were specific for human phospholipases and were incubated in blocking solution (1:200; 2 h). Every excess of primary antibody was then removed twice using wash solution for 10' and immune-reactive bands were subsequently incubated with goat anti-mouse and anti-rabbit IgG-HRP secondary antibodies (1:2,000) in blocking solution for 1 h at room temperature. Blots were washed four times for 10' (each time) in PBS and 0.05% Tween 20 solution and blotted proteins were revealed using Immobilon Western Chemiluminescent HRP Substrate (Millipore, Billerica, MA, USA) according to the manufacturer's protocol with maximum exposure time of 1'.

### Other assays

Tumor necrosis factor-α (TNFα), an inductor of sPLA2 expression [10], was measured using an ELISA kit having as detection range 23-1,500 pg/mL. FFA, the products of sPLA2 reaction, were measured using a colorimetric assay [20] having as detection range was 7-1,000 μM. Surfactant Protein-A (SP-A) was measured using an ELISA kit having as detection range 0.16/500 ng/mL [21]. These assays (Biovendor Laboratorni, Brno, Czech Republic) had coefficients of variations ≤10%.

Proteins and urea were measured, as previously described [22,23]. Serum urea values obtained during the routine clinical tests in the same day of the lavage were used to calculate the serum-to-BALF urea ratio and obtain epithelial lining fluid (ELF) concentrations.

### Biophysical study

Sixteen BALF specimens consisted of a remaining volume ≥500 μL, beyond that used for the above-described assays. These specimens were immediately frozen and sent under dry ice to the biophysical laboratory. Total native surfactant (large plus small aggregates) was purified by ultracentrifugation (100,000 g; 4°C; 1 h), used in aqueous suspension and diluted with Tris buffer (5 mM; pH 7) containing 150 mM NaCl to the final desired phospholipids concentration, without organic extraction. Phospholipids were measured as previously published [24]; detection limit was 11 μg/mL.

Surfactant activity was then evaluated using captive bubble surfactometry (CBS), as previously described [25,26]. In detail, CBS chamber was thermostated at 37°C and contained 5mM Tris-HCl (pH 7), 150 mM NaCl, and 10% sucrose. After a small air bubble (0.035-0.040 cm^3^) was formed, approximately 150 nL of surfactant (10mg/mL) was deposited below the bubble surface with a transparent capillary, as previously described [27]. Following the introduction of surfactant, changes in surface tension were monitored for 5 min (initial adsorption) from the change in the bubble shape [28]. The chamber was then sealed and the bubble was quickly (1s) expanded to 0.15 cm^3^, to record post-expansion adsorption during 5 min. Then quasi-static cycles started, where the bubble size was first reduced and then enlarged in a stepwise fashion. There was 1' delay between the four quasi-static cycles and the following dynamic cycles. During dynamic cycles the bubble was continuously compressed and expanded at a rate of 20 cycles/min.

All laboratory assays were performed in triplicate by investigators blinded to the clinical data.

### Clinical data

Clinical data, PRISM-III_24 _[29], and derived predicted mortality were recorded. Single breath static respiratory system compliance (Crs) was measured, just before the lavage, under controlled ventilation, using a passive exhalation following end-inspiratory occlusion [30]. 15' before lavage, routine gas analysis was also performed from an indwelling arterial line: PaO_2_/FiO_2 _and oxygenation index (OI= ((mean airway pressure) ^x ^FiO_2_/PaO_2_)) were recorded. Severity of ARDS was assessed using the Murray's lung injury score modified for children [30]. Finally, length of PICU stay, duration/type of ventilation, and duration of oxygen therapy were downloaded from bedside monitoring system (DIGISTAT^®^, United Medical Software, Cerbaia, FI, Italy).

### Statistics

Data were tested for normality with Shapiro-Wilk test, expressed as median (interquartile range) or mean ± standard deviation, and analyzed using Mann-Whitney, Student, χ^2^, or Fisher test, as appropriate. Spearman (ρ) correlation was also applied. Correlations were corrected adjusting for the study group (ARDS or control), or infants' weight and age, using partial correlation technique [31]: to do so, variables not normally distributed were previously subjected to Log-transformation. Statistics was performed using SPSS 15.0 for Windows (SPSS Inc., Chicago, IL, USA) and *P *values < 0.05 were considered to be significant.

## Results

### sPLA2 and other assays in BALF

**Figure 1:**
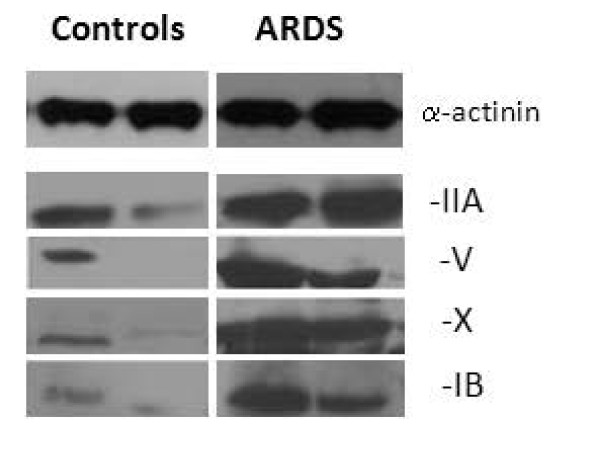
**Illustrative findings of western blotting**. Results are shown for two samples of each group, as representative. Four isotypes are expressed and secreted in BALF of ARDS infants; α-actinin is used as control. Lower amounts of sPLA2-IIA, -V, and -X were present only in some of the controls, while sPLA2-IB is nearly absent.

Western blotting (Figure 1) showed high concentrations of sPLA2-IIA and -X in 100% of ARDS patients, while high amounts of sPLA2-V and sPLA2-IB were detected in 87% and 75% of cases, respectively. Lower amount of sPLA2-IIA, -V, and -X were also observed in 64%, 50%, and 78% of controls, respectively. sPLA2-IB was nearly absent in control BALFs.

**Figure 2:**
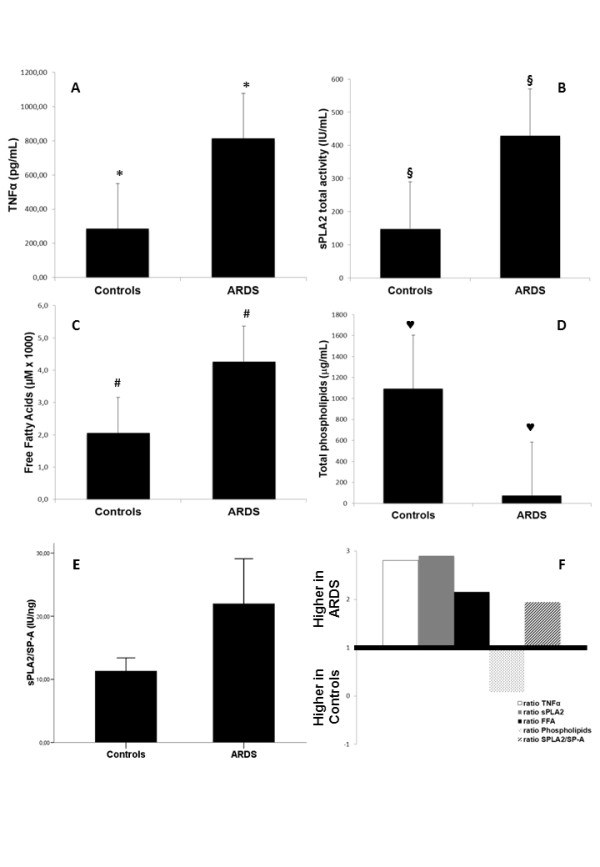
**sPLA2 and molecules related to its activity or expression in epithelial lining fluids**. Pooled data from cases and controls. TNFα (Panel A; *P = 0.04), sPLA2 total activity (Panel B; §*P *= 0.01), FFA (Panel C; #*P *= 0.026) and total phospholipids (Panel D; ♥*P *= 0.0001) are significantly different between ARDS and controls. Panel E shows sPLA2/SP-A ratio and Panel F shows results presented in the other panels, normalized as unit-free ARDS patients/controls ratio. Data are shown as medians or mean, as appropriate; T-bars represent standard error. ARDS: acute respiratory distress syndrome; FFA: free fatty acids; SP-A: Surfactant protein A; sPLA2: secretory phospholipase A2; TNFα: tumor necrosis factor-α.

Significant differences exist between cases and controls in terms of sPLA2 activity, phospholipids, TNFα, FFA (Figure 2). TNFα (814 (506-2,499) *vs*. 287 (111-1,315) pg/mL; *P *= 0.04), sPLA2 activity (430 (253-600) *vs*. 149 (61-387) IU/mL; *P *= 0.01), and FFA (4.3 (2.8-8.6) *vs*. 2 (0.8-4.6) μM; *P *= 0.026) are higher in ELF of ARDS patients than in controls. sPLA2/SP-A ratio is also higher in ARDS patients, although it does not reach significance (22 ± 30 *vs*. 11.3 ± 6 IU/ng; *P *= 0.29). Conversely, the total amount of phospholipids is lower in ELF of ARDS patients than in controls (76.5 (54-100) *vs*. 1,094 (536-2,907) μg/mL; *P *= 0.0001). Total ELF proteins were higher in ARDS patients (22 (10-29.6) mg/mL) than in controls (5.9 (4.3-25.3); *P *= 0.013).

No significant differences were found between direct and indirect ARDS in terms of ELF TNFα (800 (500-3,220) *vs*. 828 (462-2,203) pg/mL; *P *= 0.070), sPLA2 (323 (243-608) *vs*. 482 (261-580) IU/mL; *P *= 0.970), FFA (3.3 (1.6-8.8) *vs*. 4.8 (3.8-8.6) mM; *P *= 0.4), total phospholipids (92.5 (62-120.5) *vs*. 76.5 (45-82) μg/mL; *P *= 0.413), and total proteins (25 (13-65.6) *vs*. 15 (10-29) mg/mL; *P *= 0.288).

### Biochemical and biophysical effect of sPLA2

**Figure 3:**
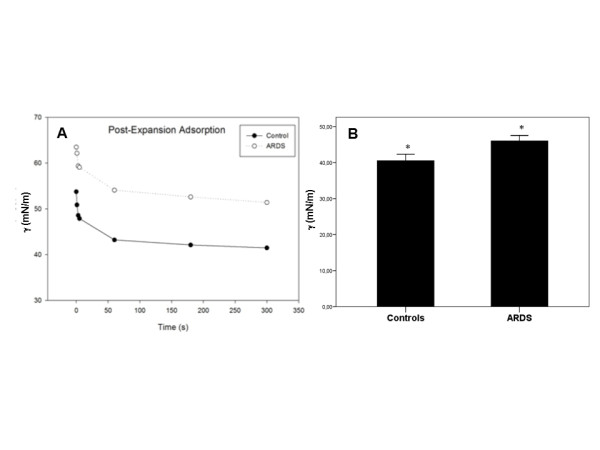
**Biophysical behavior of surfactant in terms of minimum surface tension (γ) reached by BALF samples, after 5 min of post-expansion adsorption in captive bubble surfactometry**. Panel A: Illustrative results for one sample of each group: control (black full circles) and ARDS infants (open circles). Panel B: pooled data from cases and controls: mean minimum surface tension (γ) is higher in ARDS than in controls (*P = 0.04). Data are shown as mean. T-bars represent standard error.

**Figure 4:**
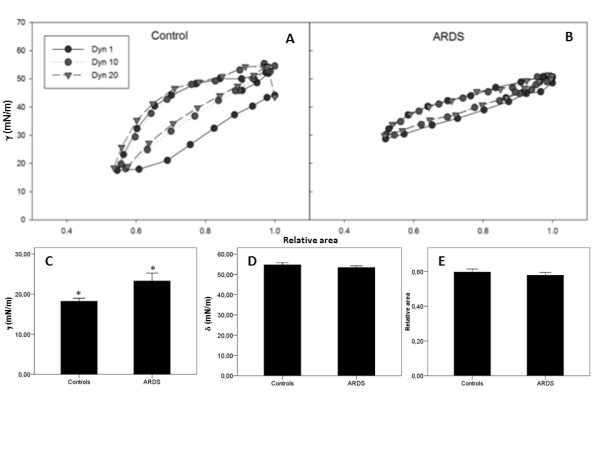
**Biophysical behavior of surfactant after repeated compression-expansion dynamic cycles in captive bubble surfactometry**. Panels A and B: illustrative results for one sample of each group in terms of minimum surface tension (γ). Dynamic cycling of control **(A) **and ARDS infants **(B)**: cycle number 1, 10, and 20 are depicted by black and grey circles and grey triangles, respectively. Panel C, D, and E: pooled data from cases and controls, after 20 compression-expansion dynamic cycles. Panel C shows mean minimum surface tension (γ) to be higher in ARDS than in controls (*P = 0.034); panels D shows mean maximum surface tension (δ); panel E shows relative area, that is the proportion of compression needed to reach γ at the end of the 20th dynamic cycle. Data are shown as mean. T-bars represent standard error.

Figures 3 and 4 describe the different interfacial behavior when comparing surfactant from cases and controls in CBS; both illustrative cases and grouped data are presented. Figure 3 shows kinetics of adsorption/reorganization of surfactant into an expanded bubble, after post-expansion adsorption from two illustrative samples (Panel A). ARDS samples do not reach the same low surface tension of control samples, indicating that surfactant from cases exhibits a deficient adsorption and reorganization upon reaching the air-bubble surface. Minimum surface tension (γ) reached by surfactant is higher in ARDS patients then in controls (45.5 ± 4.7 *vs*. 40 ± 4.5 mN/m; *P *= 0.04; Panel B).

Figure 4 shows the compression-expansion tension-area isotherms of illustrative ARDS (Panel A) and control (Panel B) samples subjected to dynamic cycling in CBS (20 cycles/min). Control sample reach γ = approximately 20 mN/m, while ARDS sample does not reach values < 30 mN/m. Grouped data show that γ is higher in ARDS patients then in controls (25.6 ± 6.1 *vs*. 18 ± 1.8 mN/m; *P *= 0.034; Panel C). Maximum surface tension (δ) is similar between cases and controls (54 ± 2.5 *vs*. 54.7 ± 2.6; *P *= 0.491; Panel D). Relative area, which is the compression rate needed to reach γ is slightly lower in cases than in controls (0.5 ± 0.04 *vs*. 0.6 ± 0.04; *P *= 0.5; Panel E).

**Figure 5:**
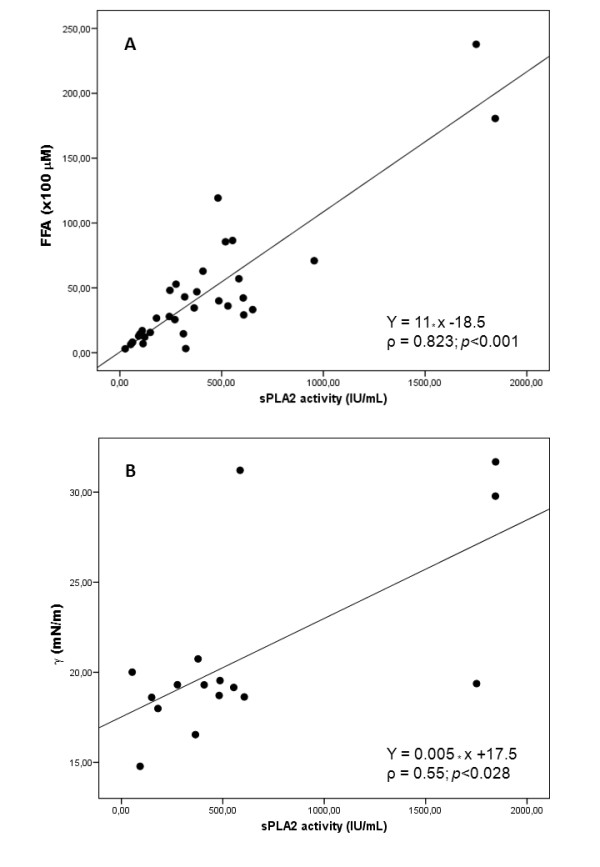
**Biochemical and biophysical correlations of sPLA2 activity in epithelial lining fluid**. Lines are drawn by the minimum square root method corresponding to the following equations: A (Y = 11*x -185); B (y = 0.005*x +17.5).

Figure 5 shows correlations between sPLA2 activity and biochemical or biophysical consequences in terms of reaction products (FFA) and minimum surface tension, respectively. There are significant correlations in ELF between sPLA2 and FFA (ρ = 0.823; *P *< 0.001) and between sPLA2 and γ (ρ = 0.55; *P *< 0.028). A significant correlation between sPLA2 and TNFα has also been found (ρ = 0.6; *P *< 0.001). These relationships remained significant when adjusting for infants' age and weight (partial correlation coefficients sPLA2-FFA: 0.88, *P *< 0.001; sPLA2-γ: 0.66, *P *= 0.005; sPLA2-TNFα: 0.57, *P *= 0.001). Analogous results were obtained adjusting for the study group (data not shown).

Table 2 reports similar significant correlations between FFA, γ, and lung mechanics and clinical severity. These correlations remained significant when adjusting for the study group (data not shown).

**Table 2:** Correlation between free fatty acids (FFA), surface tension (γ), and lung mechanics and clinical severity.

	FFA			γ		
	**ρ**	***P***	**Adj-*P***	**ρ**	***P***	**Adj-*P***

Crs	-0.46	0.009	0.011	-0.54	0.03	0.047

Murray's score	0.643	<0.001	0.004	0.57	0.021	0.035

OI	0.45	0.008	0.021	0.53	0.036	0.045

Lung mechanics are described by Crs, while clinical severity by Murray's score and OI. Spearman correlation coefficient (ρ) with its significance *p*-value. Adj-*p *is the *p*-value adjusted for infants' weight and age, using partial correlation technique.

γ: minimum surface tension; Crs: static compliance of the respiratory system; FFA: free fatty acids; OI: oxygenation index.

### Clinical outcomes

**Figure 6:**
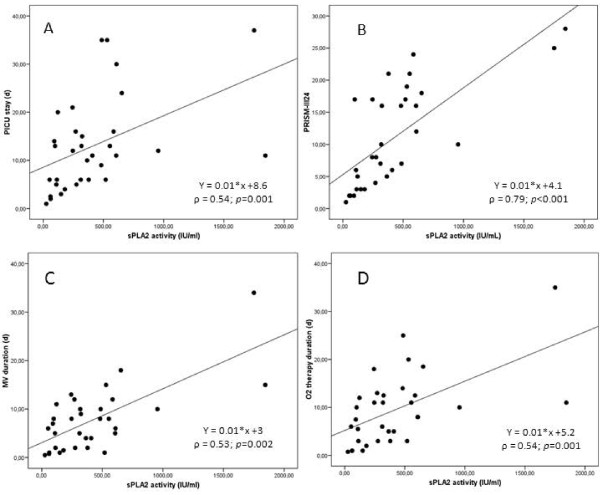
**Correlations with clinical outcomes**. Epithelial lining fluid sPLA2 activity and correlation with PICU length of stay **(A)**, PRISM-III_24 _score **(B)**, duration of mechanical ventilation **(C)**, and oxygen therapy **(D)**. Lines are regression lines drawn by the minimum square root method: A (y = 0.01*x +8.6); B (Y = 0.01*x +4.1); C (y = 0.01*x +3); D (y = 0.01*x +5.2). MV: mechanical ventilation; PICU: Pediatric intensive care unit; sPLA2: secretory phospholipase A2.

Significant correlations between sPLA2 activity and clinical outcomes are shown in Figure 6 and remained significant after adjusting for infants' weight and age. Details were as follows: sPLA2 activity and PICU stay (ρ = 0.54; *P *= 0.001; adj-*P *= 0.001), sPLA2 and PRISM-III_24 _(ρ = 0.79; *P *< 0.001; adj-*P *< 0.001), sPLA2 and duration of mechanical ventilation (ρ = 0.53; *P *= 0.002; adj-*P *< 0.001), sPLA2 and duration of oxygen therapy (ρ = 0.54; *P *= 0.001; adj-*P *= 0.001). Similar correlation exists between sPLA2 and predicted mortality rate derived from PRISM-III_24 _(ρ = 0.76; *P *< 0.001; adj-*P *< 0.001). These correlations remained significant when adjusting for study group (data not shown). No other correlations were found.

Eight ARDS patients (30%) needed rescue HFOV and they presented significantly higher sPLA2 activity in ELF (597 (367-1,476) IU/mL), as compared to ARDS patients remaining under conventional ventilation (321 (152-509) IU/mL; *P *= 0.02). γ also resulted slightly higher in ARDS patients needing HFOV than in those conventionally ventilated (25 ± 6.8 *vs*. 21 ± 5.3 mN/m; *P *= 0.47), although this does not reach significance.

## Discussion

This study shows that alveolar sPLA2 plays a role in pediatric ARDS. sPLA2 activity consisted of four distinct enzyme subtypes and may cause biochemical and biophysical consequences. In fact, sPLA2 activity seems to be downstream correlated with clinical severity and some clinical outcomes. This study partially expands the knowledge accumulated on adults and, for the first time, provides specific data for pediatric ARDS in this regard.

### sPLA2 activity and identification

sPLA2 activity is increased, as well as TNFα [10], and FFA, the product of reaction catalyzed by sPLA2. As previously reported [12], a significant correlation exists between TNFα and sPLA2, confirming the role of TNFα as enhancer of sPLA2 expression. [10] This leads to relevant consequences in terms of biochemical and biophysical effects. Total amount of phospholipids is also reduced in BALF of ARDS patients, although a qualitative alteration of the phosphilipid pool is also likely to occur (see below). Adult ARDS patients have lower levels of SP-A [21] and this protein is known to directly inhibit sPLA2-IIA [32,33]. SP-A binds sPLA2 through a direct protein-protein interaction, thus it has been expressed as a ratio [32,33]. Our data seem to suggest that SP-A could be relatively lacking and that the interaction between sPLA2 and SP-A could play a role also in pediatric ARDS patients. However, the difference in sPLA2/SP-A ratio between cases and controls is not statistically significant, possibly due to our small population size: claryfing the role of SP-A is worth to be done with a specific study. In fact, commercially available surfactants do not have SP-A and this may account for the variable efficacy of surfactant replacement therapy. [9]

sPLA2-IIA, -V, and -X are highly expressed in nearly all infants and secreted into the alveoli. These subtypes are also detected, although in lower amount, in some controls and this is consistent with animal data showing their expression in total lung extracts, as sPLA2-IIA is produced from various circulating cells [2] and sPLA2-V and -X from airway epithelial cells [34]. Presence of some amount of sPLA2 isotypes may be due to their physiologic roles as anti-infective molecules [2] or to a certain degree of inflammation. In fact, our controls were intubated infants and some ventilation-induced inflammation is unavoidable. While the importance of sPLA2-IIA had been already highlighted in ARDS [5,6], sPLA2-V and -X were only suspected to play a role, given the animal data available so far. These studies showed that sPLA2-V expression in mice lung is associated with surfactant hydrolysis [35] and gene targeted mice lacking sPLA2-V presented a milder form of acute lung injury with lower inflammation [36]. Similarly, sPLA2-X can efficiently hydrolyse surfactant phospholipids *in vitro *and this activity is also inhibited by SP-A [2,33]. Our findings in human infants with ARDS seem to confirm a role for sPLA2-V and -X. A propeptide of sPLA2-IB had been detected in serum of adult ARDS patients but not in controls [37]: this is consistent with our data showing presence of sPLA2-IB in 75% of patients. Given the widespread tissue distribution of sPLA2-IB [1,2], it is possible that this subtype arrives to the lung after production elsewhere or from circulating inflammatory cells reaching the lung. Alternatively, sPLA2-IB may be locally triggered by sPLA2-IIA, since a specific cross-talk exists between the two subtypes [38]. These data are relevant, as many sPLA2 inhibitors are now under advanced investigation and carry different inhibitory potency against the four described subtypes [11].

### Biochemical and biophysical effect of sPLA2

There is a deficient adsorption and re-organization of material into the air/water interface leading to higher surface tension in ARDS infants, both after the post-expansion adsorption and during the breathing-mimicking, compression-expansion dynamic cycling. The different shape of the loops is also consistent with these data. Since CBS was carried out at the same phospholipid concentration, it is the quality of surfactant that seems to be affected in ARDS. This qualitative alteration has been already described in adult ARDS patients [39] and may be related to the activity of the various sPLA2 subclasses secreted into the alveoli. Concomitantly, the presence of other inflammatory proteins and higher amounts of neutral lipids may also be partially responsible for surfactant alteration [40,41].

Increased surface tension at the end of the compression has already been described in adult ARDS and supposed to cause atelectasis and lung collapse [42]. This is confirmed by our findings, as minimal surface tension is significantly correlated to Crs and to gas exchange impairment. The latter two have been linked with sPLA2 in animal studies and in adults with ARDS [3-6]. Interestingly, not only surface tension, but also FFA, correlate with these physiopathologic variables. It has been demonstrated that the two products of sPLA2 reaction, lysophospholipids and FFA, are potent inhibitors of surfactant biophysical activity [42]. Conversely, FFA are precursors of various proinflammatory mediators: thus, the effect on lung compliance and oxygenation may be not only due to the direct lypolitic activity of sPLA2 on surfactant phospholipids, but also to lysophospholipids and FFA inhibiting the remaining surfactant and to the additional contribution of other FFA-derived inflammatory molecules. Consistently, the Murray's lung injury score, that globally describe the clinical severity (taking into accout Crs, oxygenation deficit, and other variables), also correlates with both surface tension and FFA. We must admit that correlations shown in Table 2 are statistically significant but lower than others, thus a strong and direct relationship cannot be assumed just from these findings. Given the biological background, a link is likely to exist between the analyzed variables, but it is also possible that other factors are involved in these relationships influencing the results [40,41].

Minimal surface tension produced upon cycling of control samples under our experimental conditions are higher than those required to stabilize the lungs. In principle, a good functional surfactant film should produce tensions <5 mN/m when compressed in CBS [43]. It is possible that sampled surfactants are particularly enriched in material from the upper airways, with more limited surface activity that surfactant freshly secreted by the alveolar epithelium. It is also possible that mechanical ventilation might induce an inhibitory effect, both in controls and in ARDS patients, affecting the minimal tension reached by the relatively diluted concentrations of surfactant tested here. Finally, it is also conceivable that differences in minimal tensions between controls and ARDS patients could be maximized if tested at higher surfactant concentrations. Unfortunately, the limited sample volume available from infants prevented the assay at concentrations >10 mg/mL. Anyway, all samples were assessed at equivalent phospholipid concentrations, indicating that the differences found between controls and ARDS patients are indicative of an actual alteration of surfactant function.

### Clinical outcomes

Raised sPLA2 activity, through the increment in inflammation and surface tension that impairs lung mechanics and oxygenation, might finally have relevant clinical consequences. In fact, as secondary study aim, we found sPLA2 activity to be significantly correlated with PICU stay, duration of mechanical ventilation, and oxygen therapy. Our population mortality is consistent with other reports [7]. Since pediatric ARDS has a relatively low incidence and mortality, this latter is not a feasible clinical endpoint, while shorter respiratory support is an accepted alternative outcome resulting from decreasing inflammation [7]. Similarly, PICU stay is an alternative, as it may impact on mortality through the increased risk of nosocomial infections and on the general burden of care. sPLA2 activity is also correlated with the PRISM-III_24 _and its predicted mortality. Similar correlation with predicted mortality was described in adults using a different score commonly applied in adult critical care [6]. About 30% to 40% of PICUs worldwide are using HFOV as rescue method for respiratory support in children unresponding to conventional ventilation [7] and since we have a strict protocol for rescue HFOV, we were able to analyze this as a secondary additional outcome. Consistently with the other findings, babies needing HFOV turned out to have higher sPLA2 levels. Rescue HFOV may use higher Pāw to recruit alveoli in more stiff and collapsed lungs; thus, it seems logical that patients with higher sPLA2 activity have more diseased lungs and require such technique to be ventilated.

There are some study limitations and some questions still to be answered. Ours is a relatively small population and larger cohorts may be required to accurately study the correlation between sPLA2 and clinical outcomes. Scarce data are available about lung function of ARDS surviving-children [44], although a spectrum of different consequences is conceivable. A larger study is required to clarify if sPLA2 over-activity may predispose to chronic lung diseases in ARDS survivors or if there is any alteration of pulmonary function test later on.

Given the small population size and the descriptive nature of the study, limited conclusions can be generalized to other infants: other confounding factors should be taken into account. In detail, genomic population surveys including sPLA2 and other genes must be performed in order to verify the predisposition for ARDS. Our international project [13] already included a genetic study on the sPLA2 isoforms polymorphisms: this study is still in progress and provisional results have been presented elsewhere [45].

Our patients have been sampled only once at the study enrolment and we cannot provide data during the ARDS course. Around 58% of our cases are represented by infection-related ARDS: different triggering conditions might influence some results.

Moreover, while the interaction between SP-A and sPLA2-IIA is known [32,33], there are no data about the other sPLA2 isoforms. Recently, Surfactant Protein-B (SP-B) resulted able to inhibit phospholipid hidrolysis induced by both sPLA2-IB and -IIA [46]. This effect is not due to a direct enzymatic inhibition and a synergistic effect between different (hydrophobic and hydrophilic) surfactant proteins has been hypothesized [46]. The interaction between surfactant proteins and distinct sPLA2 deserves to be studied, as inflammation and clinical consequences could be more strictly related to the complex biological interactions between various bioactive molecules, rather than by the effect of a single one.

It would be also interesting to study serially infants with repeated lavages during the ARDS course: this may give clues about the progression of inflammation. It may also indicate how sPLA2 might influence the function of alveolar macrophages or the production and activity of different surfactant proteins. This requires a specific study not easy to be done in such small critically ill patients, where lavages procedures are not totally harmless and cell recovery is often unsatisfactory.

Finally, different subtypes profile could have relevant consequences on clinical pictures and future treatment approaches: we identified various sPLA2 subtypes, but we were not able to measure their relative amount or activity. In fact, we do not have available densitometry, and besides, a more precise activity measurement or separation should have been performed. This is more difficult, given the extreme sequence similarity of sPLA2 isoforms and we mean to work on this in a future study. Moreover, an increased sPLA2 activity is likely to be caused by an increased production of all (or some) isotypes. However, we cannot exclude that there may be some protein modifications increasing enzymatic activity without increasing its expression, but this has never been found till now, whereas increment in some subtypes (sPLA2-IIA and -IB) has been demonstrated in adult ARDS patients [5,6,37].

## Conclusions

Total sPLA2 activity is raised in infants with ARDS and constituted of four enzyme subtypes. sPLA2 is correlated with some inflammatory mediators and surface tension. These two are correlated in their turn with lung compliance and oxygenation impairment. Relevant clinical consequences might be caused, as sPLA2 seems correlated with predicted mortality rate, longer PICU stay, mechanical ventilation, and oxygen support. These findings describe sPLA2 as a potential target in infants with ARDS.

## Key messages

• sPLA2 is constituted of at least four isotypes during pediatric ARDS.

• sPLA2 is correlated with pediatric ARDS physiopathology (surface tension, lung compliance, oxygenation impairment).

• sPLA2 is correlated with clinical outcomes in pediatric ARDS (predicted mortality, PICU stay, duration of mechanical ventilation, and oxygen therapy).

## List of abbreviations

ARDS: acute respiratory distress syndrome; BALF: broncho-alveolar lavage fluids; CBS: captive bubble surfactometry; Crs: static compliance of the respiratory system; Dyn1, Dyn10, Dyn20: measures after 1, 10, or 20 compression-expansion dynamic cycles; ELF: epithelial lining fluid; FFA: free fatty acids; GBS: Group B streptococcus; HFOV: high frequency oscillatory ventilation; MV: mechanical ventilation; OI: oxygenation index; Pāw: mean airway pressure; PEEP: positive end-expiratory pressure; PICU: pediatric intensive care units; RSV: respiratory syncitial virus; SAH: sub-arachnoid hemorrhage; SatO_2_: arterial oxygen saturation; SP-A: surfactant protein A; sPLA2: secretory phospholipase A2; TNFα: tumor necrosis factor-alfa; V_T_: tidal volume; γ: minimum surface tension; δ: maximum surface tension.

## Competing interests

The authors declare that they have no competing interests.

## Authors' contributions

DDL, EC, and JPG designed the study and the experiments in detail; MP, GC, ES, and LG performed the BAL procedures, collected and interpreted the clinical data; AM, ELR, FV, LG, ME, and EC performed the experiments and interpreted their data; DDL, GC, MA, MP, JPG, and AM performed the complete data analysis and overall interpretation; DDL, ELR, and EC drafted the manuscript; AM, FV, ES, EC, GC, MP, MA, ME, and JPG revised the manuscript for important intellectual content. All authors fulfilled the authorship criteria and all authors read and approved the final manuscript.

## Authors' information

DLD and ED are the coordinators of the international Study group on Secretory Phospholipase in Paediatrics (SSPP).

## References

[B1] Lambeau G Gelb MH Biochemistry and physiology of mammalian secreted phospholipases A2 Annu Rev Biochem 2008 17 21.1 21.26 10.1146/annurev.biochem.76.062405.154007 18405237

[B2] Kitsiouli E Nakos G Lekka ME Phospholipases A2 subclasses in acute respiratory distress syndrome Biochim Biophys Acta 2009 17 1241 1248 10.1016/j.bbadis.2009.06.007 19577642

[B3] Furue S Kuwabara K Mikawa K Nishina K Shiga M Maekawa N Ueno M Chikazawa Y Ono T Hori Y Matsukawa A Yoshinaga M Obara H Crucial role for group II-phospholipase A2 in oleic acid-induced acute lung injury in rabbits Am J Resp Crit Care Med 1999 17 1241 1248 10.1164/ajrccm.160.4.9812042 10508821

[B4] Furue S Mikawa K Nishina K Shiga M Ueno M Tomita Y Kuwabara K Teshirogi I Ono T Hori Y Matsukawa A Yoshinaga M Obara H Therapeutic time-window of a group IIA phospholipase inhibitor in rabbit acute lung injury: correlation with lung surfactant protection Crit Care Med 2001 17 1241 1248 10.1097/00003246-200104000-00004 11373455

[B5] Kim DK Fukuda T Thompson BT Cockrill B Hales C Bonventre JV Bronchoalveolar lavage fluid phospholipase A2 activities are increased in human adult respiratory distress syndrome Am J Physiol 1995 17 L109 118 7631805 10.1152/ajplung.1995.269.1.L109

[B6] Nakos G Kitsiouli E Hatzidaki E Koulouras V Touqui L Lekka ME Phospholipases A2 and platelet activating-factor acetylhydrolase in patients with acute respiratory distress syndrome Crit Care Med 2005 17 1241 1248 10.1097/01.ccm.0000158519.80090.74 15818104

[B7] Randolph AG Management of acute lung injury and acute respiratory distress syndrome i children Crit Care Med 2009 17 1241 1248 10.1097/CCM.0b013e3181aee5dd 19531940

[B8] Khemani RG Newth CJL The design of future pediatric mechanical ventilation trials for acute lung injury Am J Resp Crit Care Med 2010 17 1241 1248 10.1164/rccm.201004-0606CI PMC3029934 20732987

[B9] De Luca D Cogo P Zecca E Piastra M Pietrini D Tridente A Conti G Carnielli VP Intrapulmonary drug administration in neonatal/pediatric critical care: a comprehensive review Eur Resp J 2011 17 1241 1248 10.1183/09031936.00024910 21357925

[B10] Touqui L Arbibe L A role for phospholipase A2 in ARDS pathogenesis Mol Med Tod 1999 17 1241 1248 10.1016/s1357-4310(99)01470-7 10366819

[B11] Magrioti V Kokotos G Phospholipase A2 inhibitors as potential therapeutic agents for the treatment of inflammatory diseases Expert Opin Ther Pat 2010 17 1241 1248 10.1517/13543770903463905 20021282

[B12] De Luca D Minucci A Cogo P Capoluongo ED Conti G Pietrini D Carnielli VP Piastra M Secretory phospholipase A2 pathway during pediatric ARDS: a preliminary study Pediatr Crit Care Med 2011 17 e20 e24 10.1097/PCC.0b013e3181dbe95e 20351613

[B13] De Luca D Capoluongo ED Rigo V for the Study group on Secretory Phospholipase in Paediatrics (SSPP) Secretory phospholipase A2 pathway in various types of lung injury in neonates and infants: a multicentre translational study BMC Pediatrics 2011 17 101 10.1186/1471-2431-11-101 22067747 PMC3247178

[B14] Bernard GR Artigas A Brigham KL Carlet J Falke K and the Consensus Committee American-European Consensus Conference on ARDS Definition, mechanisms, relevant outcomes and clinical trial coordination Am J Respir Crit Care Med 1994 17 1241 1248 10.1164/ajrccm.149.5.8173765 7509706

[B15] Ventre KM Arnold JH High frequency oscillatory ventilation in acute respiratory failure Pediatr Respir Rev 2004 17 1241 1248 10.1016/j.prrv.2004.07.002 15531258

[B16] Randolph AG Wypij D Venkataraman ST Hanson JH Gedeit RG Meert KL Luckett PM Forbes P Lilley M Thompson J Cheifetz IM Hibberd P Wetzel R Cox PN Arnold JH Pediatric Acute Lung Injury and Sepsis Investigators (PALISI) Network Effect of mechanical ventilator weaning protocols on respiratory outcomes in infants and children: A randomized controlled trial JAMA 2002 17 1241 1248 10.1001/jama.288.20.2561 12444863

[B17] European Respiratory Society Task Force on bronchoalveolar lavage in children Bronchoalveolar lavage in children Eur Respir J 2000 17 217 231 10.1183/09031936.00.15121700 10678650

[B18] De Luca D Minucci A Tripodi D Piastra M Pietrini D Zuppi C Conti G Carnielli VP Capoluongo E Role of distinct phospholipases A2 and their modulators in meconium aspiration syndrome in human neonates Intensive Care Med 2011 17 1241 1248 10.1007/s00134-011-2243-z 21567110

[B19] Reynolds LJ Hughes LL Dennis EA Analysis of human synovial fluid phospholipase A2 on short chain phosphatidylcholine-mixed micelles: development of a spectrophotometric assay suitable for a microtiter plate reader Anal Biochem 1992 17 1241 1248 10.1016/0003-2697(92)90160-9 1514686

[B20] Matsubara C Nishikawa Y Yoshida Y Takamura K A spectrophotometric method for the determination of free fatty acid in serum using acyl-coenzyme A synthetase and acyl-coenzyme A oxidase Anal Biochem 1983 17 1241 1248 10.1016/0003-2697(83)90659-0 6869794

[B21] Greene KE Wright JR Steinberg KP Ruzinski JT Caldwell E Wong WB Hull W Whitsett JA Akino T Kuroki Y Nagae H Hudson LD Martin TR Serial changes in surfactant-associated proteins in lung and serum before and after onset of ARDS Am J Respir Crit Care Med 1999 17 1241 1248 10.1164/ajrccm.160.6.9901117 10588595

[B22] Bradford M A rapid and sensitive method for the quantitation of microgram quantities of protein utilizing the principle of protein-dye binding Anal Biochem 1976 17 1241 1248 10.1016/0003-2697(76)90527-3 942051

[B23] Capoluongo E Ameglio F Lulli P Minucci A Santonocito C Concolino P Di Stasio E Boccacci S Vendettuoli V Giuratrabocchetta G De Cunto A Tana M Romagnoli C Zuppi C Vento G Epithelial lining fluid free IGF-I-to-PAPP-A ratio is associated with bronchopulmonary dysplasia in preterm infants Am J Physiol Endocrinol Metab 2007 17 E308 E313 16954333 10.1152/ajpendo.00251.2006

[B24] Rouser G Siakotos AN Fleischer S Quantitative analysis of phospholipids by thin-layer chromatography and phosphorus analysis of spots Lipids 1966 17 1241 1248 10.1007/BF02668129 17805690

[B25] Schuürch S Bachofen H Goerke J Possmayer F A captive bubble method reproduces the in situ behavior of lung surfactant monolayers J Appl Physiol 1989 17 1241 1248 10.1152/jappl.1989.67.6.2389 2606846

[B26] Goómez-Gil L Schuürch D Goormaghtigh E Peórez-Gil J Pulmonary surfactant protein SP-C counteracts the deleterious effects of cholesterol on the activity of surfactant films under physiologically relevant compression-expansion dynamics Biophysical J 2009 17 1241 1248 10.1016/j.bpj.2009.08.045 PMC2776298 19917227

[B27] López-Rodríguez E Ospina OL Echaide M Taeusch TW Pérez-Gil J Exposure to polymers reverses inhibition of pulmonary surfactant by serum, meconium, or cholesterol in the captive bubble surfactometer Biophysical J 2012 17 1241 1248 10.1016/j.bpj.2012.08.024 PMC3471484 23062337

[B28] Schoel WM Schuürch S Goerke J The captive bubble method for the evaluation of pulmonary surfactant: surface tension, area, and volume calculations Biochim Biophys Acta 1994 17 1241 1248 10.1016/0304-4165(94)90169-4 8068714

[B29] Pollack MM Patel KM Ruttimann UE PRISM-III: an updated pediatric risk of mortality score Crit Care Med 1996 17 1241 1248 10.1097/00003246-199605000-00004 8706448

[B30] Newth CJ Stretton M Deakers TW Hammer J Assessment of pulmonary function in the early phase of ARDS in pediatric patients Ped Pulmonol 1997 17 1241 1248 10.1002/(sici)1099-0496(199703)23:3<169::aid-ppul1>3.0.co;2-j 9094724

[B31] Norusis M SPSS 13.0 advanced statistical procedures companion 2004 Prentice Hall, NJ: Upper Saddle-River

[B32] Arbibe L Koumanov K Vial D Rougeot C Faure G Havet N Longacre S Vargaftig BB Béréziat G Voelker DR Wolf C Touqui L Generation of lyso-phospholipids from surfactant in acute lung injury is mediated by type-II phospholipase A2 and inhibited by a direct surfactant protein A-phospholipase A2 protein interaction J Clin Invest 1998 17 1241 1248 10.1172/JCI3236 PMC509098 9739049

[B33] Chabot S Koumanov K Lambeau G Gelb MH Balloy V Chignard M Whitsett JA Touqui L Inhibitory effects of surfactant protein-A on surfactant phosoholipid hydrolysis by secreted phospholipases A2 J Immunol 2003 17 1241 1248 10.4049/jimmunol.171.2.995 12847272

[B34] Seeds MC Jones KA Duncan Hite R Willingham MC Borgerink HM Woodruff RD Bowton DL Bass DA Cell-specific expression of group × and group V secretory phospholipase A2 in human lung airway epithelial cells Am J Respir Cell Mol Biol 2000 17 1241 1248 10.1165/ajrcmb.23.1.4034 10873151

[B35] Ohtsuki M Taketomi Y Arata S Masuda S Ishikawa Y Ishii T Takanezawa Y Aoki J Arai H Yamamoto K Kudo I Murakami M Transgenic expression of group V, but not group X, secreted phospholipase A2 in mice leads to neonatal lethality because of lung dysfunction J Biol Chem 2006 17 1241 1248 17008322 10.1074/jbc.M607975200

[B36] Muñoz NM Meliton AY Meliton LN Dudek SM Leff AR Secretory group V phospholipase A2 regulates acute lung injury and neutrophilic inflammation caused by LPS in mice Am J Physiol Lung Cell Mol Physiol 2009 17 L879 L887 10.1152/ajplung.90580.2008 19286925 PMC2692807

[B37] Rae D Porter J Beechey-Newman N Sumar N Bennett D Hermon-Taylor J Type I phospholipase A2 propeptide in acute lung injury Lancet 1994 17 1241 1248 10.1016/S0140-6736(94)90744-7 7968121

[B38] Kishino J Ohara O Nomura K Kramer RM Arita H Pancreatic-type phospholipase A2 induces group II-phospholipase A2 expression and prostaglandin biosynthesis in rat mesangial cells J Biol Chem 1994 17 1241 1248 8106488

[B39] Günther A Ruppert C Schmidt R Markart P Grimminger F Walmrath D Seeger W Surfactant alteration and replacement in acute respiratory distress syndrome Respir Res 2001 17 1241 1248 10.1186/rr86 PMC64803 11737935

[B40] Casals C Varela A Ruano ML Valiño F Pérez-Gil J Torre N Jorge E Tendillo F Castillo-Olivares JL Increase of C-reactive protein and decrease of surfactant protein A in surfactant after lung transplantation Am J Respir Crit Care Med 1998 17 1241 1248 10.1164/ajrccm.157.1.9611106 9445277

[B41] Markart P Ruppert C Wygrecka M Colaris T Dahal B Walmrath D Harbach H Wilhelm J Seeger W Schmidt R Guenther A Patients with ARDS show improvement but not normalisation of alveolar surface activity with surfactant treatment: putative role of neutral lipids Thorax 2007 17 1241 1248 10.1136/thx.2006.062398 PMC2117258 17287298

[B42] Günther A Siebert C Schmidt R Ziegler S Grimminger F Yabut M Temmesfeld B Walmrath D Morr H Seeger W Surfactant alterations in severe pneumonia, acute respiratory distress syndrome and cardiogenic lung edema Am J Respir Crit Care Med 1996 17 1241 1248 10.1164/ajrccm.153.1.8542113 8542113

[B43] Hite RD Seeds MC Jacinto RB Grier BL Waite BM Bass DA Lysophospholipid and fatty acid inhibition of pulmonary surfactant: non-enzymatic models of phospholipase A2 surfactant hydrolysis Biochim Biophys Acta 2005 17 1241 1248 10.1016/j.bbamem.2005.10.014 16376294

[B44] Hummer J ARDS - Long term follow up Paediatr Resp Rev 2006 17 S192 S193 10.1016/j.prrv.2006.04.205 16798560

[B45] De Luca D Minucci A Gentile L Piastra M Antonelli M Conti G Capoluongo ED Association between secretory phospholipase A2 subtype V (PLA2G5) genotype and acute respiratory distress in infants [abstract] Arch Dis Child 2012 Suppl 2 A16

[B46] Hite RD Grier BL Waite BM Veldhuizen RA Possmayer F Yao LJ Seeds MC Surfactant protein B inhibits secretory phospholipase A2 hydrolysis of surfactant phospholipids Am J Physiol Lung Cell Mol Physiol 2012 17 L257 265 10.1152/ajplung.00054.2011 22037357 PMC3349360

